# P-1979. Localized Nasal Mucosal SARS-CoV-2 Antibody Responses and Neutralization Potential based on Exposure History

**DOI:** 10.1093/ofid/ofae631.2137

**Published:** 2025-01-29

**Authors:** Minjun Kim, Jason S Chwa, Yunho Shin, Yesun Lee, Wesley A Cheng, Jaycee Jumarang, Jeffrey Bender, Pia S Pannaraj

**Affiliations:** University of California San Diego, San Diego, California; University of Southern California, Los Angeles, California; Children’s Hospital Los Angeles, Los Angeles, California; University of California San Diego, San Diego, California; University of California San Diego, San Diego, California; University of California San Diego, San Diego, California; Children's Hospital Los Angeles, Los Angeles, California; University of California San Diego, San Diego, California

## Abstract

**Background:**

SARS-CoV-2-specific mucosal antibodies inhibit viral replication and infection within the upper respiratory tract. We investigated nasal secretory IgA (SIgA) induction differences by exposure history and protection against subsequent infection.

**Figure:**
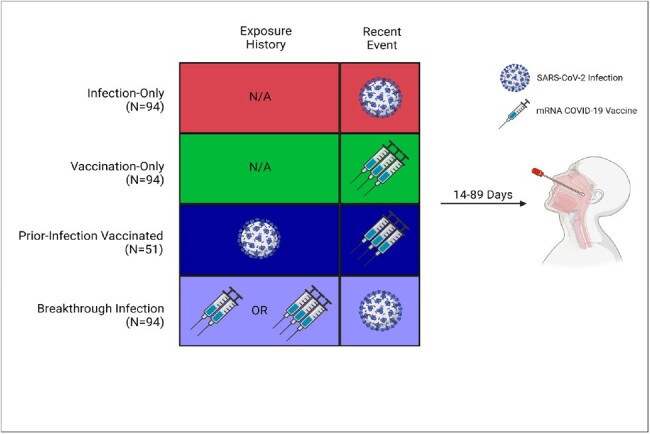

Visual schematic of exposure groups.

**Methods:**

Between June 2020 and February 2024, nasopharyngeal swab (NPS) samples were collected 14-89 days following recent infection or vaccination. SARS-CoV-2-specific nasal SIgA adjusted for total IgA (SIgA_adj_) and nasal neutralization activity were measured using ELISA and a surrogate virus neutralization test kit, respectively. Subsequent infection risk was compared in participants stratified by mucosal antibody levels using a Cox proportional hazards model.

Figure 2

**Figure:**
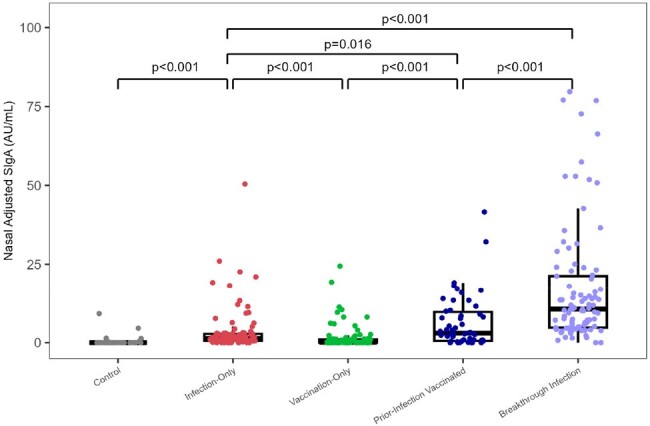

SARS-CoV-2 specific nasal secretory IgA adjusted for total IgA (SIgAadj) by exposure group compared using Wilcoxon rank sum tests.

**Results:**

NPS were collected from participants with SARS-CoV-2 infection only (n=94), vaccination only (n=94), infection then vaccination (n=51), and breakthrough infection (n=94) (Fig 1). Vaccinated individuals received 3 doses of BNT162b2 (n=199, 82.7%) or mRNA-1273 (n=40, 17.3%). The mean age was 28.2 years (range 0.4-75.1), and 197 (59.2%) were female. Individuals with breakthrough infection after vaccination mounted the highest nasal SIgA_adj_ response compared to those with infection-only (median 10.8 vs. 1.3 AU/mL*, P* < 0.001), vaccination-only (median 0.2 AU/mL, P< 0.001), or prior infection then vaccinated (median 3.2 AU/mL, *P* = 0.016) (Fig 2). Neutralization activity correlated with nasal SIgA_adj_ (ρ = 0.48, *P* < 0.001). If vaccination was the most recent exposure, SIgA did not correlate with serum IgG (ρ = 0.08, *P* = 0.3). Forty new SARS-CoV-2 infections occurred during the 12-month follow-up period (Fig 3). In a multivariable analysis to account for age, gender, and serum IgG, participants with the lowest quartile of nasal SIgA_adj_ levels had 5.8 times higher odds of infection than those with the higher three quartiles of nasal SIgA_adj_ levels within 3 months (*P* = 0.005) (Table 1).

Figure 3

**Figure:**
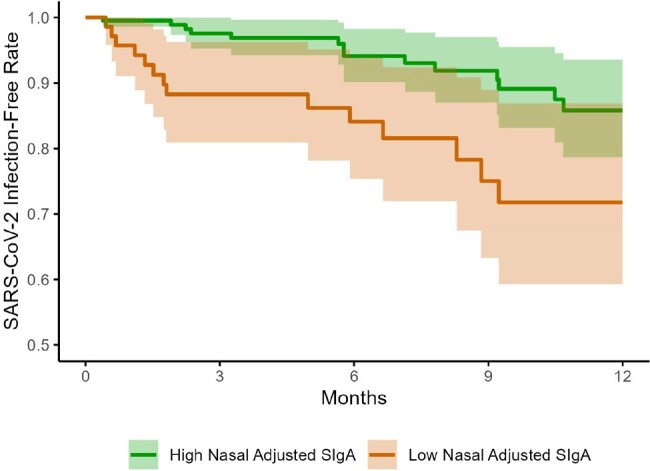

Kaplan-Meier survival curves of time to subsequent SARS-CoV-2 infection in participants stratified by SARS-CoV-2-specific nasal adjusted SIgA upper 75% quantiles (high) vs. lower 25% quartile (low) over 12 months. P = 0.004 for 0-3 months and P = 0.029 for 0-12 months.

**Conclusion:**

Our data demonstrate the protective role of nasal SIgA in lowering SARS-CoV-2 infection risk. Hybrid immunity induced the most robust mucosal response, suggesting a mucosal vaccine boost following intramuscular priming may help maximize mucosal immunity to prevent infection and community transmission.

**Figure:**
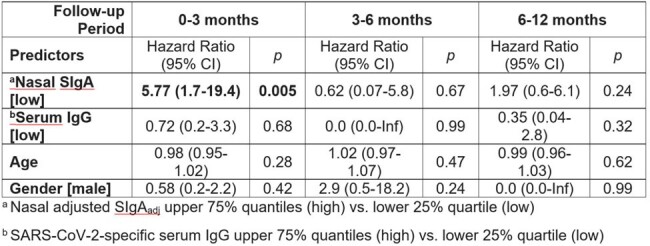

Predictors of subsequent infection within 0-3, 3-6, and 6-12 months using Cox proportional hazards regression model.

**Disclosures:**

Pia S. Pannaraj, MD, MPH, AstraZeneca: Grant/Research Support|Pfizer: Grant/Research Support

